# Response rate and long-term survival in patients with advanced melanoma: data from the prospective cohort study gem-1801

**DOI:** 10.1007/s12094-025-04098-3

**Published:** 2025-11-28

**Authors:** Enrique Espinosa, Miguel-Ángel Berciano-Guerrero, Eva Muñoz-Couselo, Teresa Puértolas, José Luis Manzano, Ainara Soria, Pablo Ayala de Miguel, Guillermo Crespo, Lourdes Gutiérrez Sanz, Pablo Cerezuela-Fuentes, Carlos Aguado de la Rosa, Margarita Majem, Almudena García Castaño, Alfonso Berrocal, Francisco Ramon García Arroyo, María Rodríguez de la Borbolla, Lorena Bellido, Javier Medina Martínez, Luis Antonio Fernández, Alfonso Martin Carnicero, Rafael López Castro, Aida Bujosa Rodríguez, Mª José Lecumberri, José Carlos Villa Guzmán, Begoña Campos Balea, Joaquín Fra Rodríguez, Javier Valdivia Bautista, Victor Navarro Pérez, Karmele Mujika, Mónica Corral Subias, Berta Hernández, Salvador Martín Algarra, Iván Márquez-Rodas

**Affiliations:** 1 Universidad Autónoma de Madrid, School of Medicine—Hospital Universitario La Paz, CIBERONC, Madrid, Spain; 2 Medical Oncology Department, Hospital Regional Universitario (HRU), Instituto de Investigaciones Biomédicas de Málaga (IBIMA), Málaga, Spain; 3 Medical Oncology Department, Hospital Universitari Vall d’Hebron y Vall d’Hebron Institute of Oncology (VHIO), Barcelona, Spain; 4 Medical Oncology Department, Hospital Universitario Miguel Servet, Zaragoza, Spain; 5 Medical Oncology Department, Instituto Catalán de Oncología, ICO-Badalona, H. Germans Trias i Pujol, Badalona, Spain; 6 Medical Oncology Department, Hospital Universitario Ramón y Cajal, Madrid, Spain; 7 Medical Oncology Department, Hospital Universitario San Pedro de Alcántara, Cáceres, Spain; 8 Medical Oncology Department, Hospital Universitario de Burgos, Burgos, Spain; 9 Medical Oncology Department, Hospital Universitario Puerta de Hierro, Madrid, Spain; 10 Medical Oncology Department, Instituto Murciano de Investigación Sanitaria (IMIB). Hospital Clínico Universitario Virgen de la Arrixaca, Ciudad de Murcia, Spain; 11 Medical Oncology Department, Hospital Universitario Clínico San Carlos, Madrid, Spain; 12 Medical Oncology Department, Hospital de la Santa Creu i Sant Pau, Barcelona, Spain; 13 Medical Oncology Department, Hospital Universitario Marqués de Valdecilla, Santander, Spain; 14 Medical Oncology Department, Hospital General Universitario Valencia, Valencia, Spain; 15 Medical Oncology Department, Complejo Hospitalario Universitario de Pontevedra, Pontevedra, Spain; 16 Medical Oncology Department, Hospital Universitario de Valme, Sevilla, Spain; 17 Medical Oncology Department, SAL), Hospital Universitario Salamanca, Instituto de Investigación Biomédica de Salamanca (IB, Salamanca, Spain; 18 Medical Oncology Department, Hospital Universitario de Toledo, Toledo, Spain; 19 Medical Oncology Department, Hospital Universitari Parc Taulí de Sabadell, Sabadell, Spain; 20 Medical Oncology Department, Hospital San Pedro de La Rioja, Logroño, Spain; 21 Medical Oncology Department, Hospital Clínico Universitario de Valladolid, Valladolid, Spain; 22 Medical Oncology Department, Hospital Son Llátzer, Palma, Spain; 23 Medical Oncology Department, Hospital Universitario de Navarra, Pamplona, Navarra Spain; 24 Medical Oncology Department, Hospital General de Ciudad Real, Ciudad Real, Spain; 25 Medical Oncology Department, Hospital Lucus Augusti, Lugo, Spain; 26 Medical Oncology Department, Hospital Universitario Rio Hortega, Valladolid, Spain; 27 Medical Oncology Department, Hospital Universitario Virgen de las Nieves, Granada, Spain; 28 Medical Oncology Department, Hospital Universitario Costa del Sol, Marbella, Spain; 29 Medical Oncology Department, Onkologikoa Fundazioa, Donosti, Spain; 30 Medical Oncology Department, Hospital Clínico Universitario Lozano Blesa, Zaragoza, Spain; 31 Medical Oncology Department, Hospital Universitario de la Princesa, Madrid, Spain; 32 Medical Oncology Department, Clínica Universidad de Navarra, Pamplona, Spain; 33 Medical Oncology Department, Hospital General Universitario Gregorio Marañón, Madrid, Spain

**Keywords:** Melanoma, Real-world, Immunotherapy, Targeted therapy, Chemotherapy, Long-term survival

## Abstract

**Purpose:**

Anti-PD-1-based immunotherapy and targeted therapies (TT) are the current standard of care for patients with advanced melanoma. However, some patients die in less than a year from diagnosis and others become long-survivors. An accurate description of systemic treatments and patients’ characteristics of long-term survivors are needed to guide treatment decisions in clinical practice.

**Methods:**

GEM-1801 is a multi-cohort prospective study from the Spanish Multidisciplinary Melanoma Group that includes patients with resectable stage III or unresectable stage III/IV melanoma. Patients surviving at least 5 years and patients surviving less than 1 year since diagnosis were selected. Baseline characteristics and first and second-line systemic treatments are described and compared between groups.

**Results:**

From Aug 2018 to Dec 2023, 60 patients with long-term survival and 216 patients with short-term survival were included. Long-term survivors received immunotherapy (65%), including anti-PD-1 in 86% and combination of an anti-PD-1 and anti-CTLA-4 in 14%; and TT (35%). The Objective Response Rate (ORR) was 76% for anti-PD-1, 80% for combination immunotherapy and 83% for TT. Short-term survivors received immunotherapy (58%), including anti-PD-1 (70%), anti-CTLA-4 (1%), and anti-PD-1 plus anti-CTLA-4 (29%); TT (36%); and chemotherapy (2%). ORR was 7% for anti-PD-1, 0% for anti-CTLA-4, 14% for combination immunotherapy, 53% for TT and 0% for chemotherapy.

**Conclusions:**

Response to first or second-line systemic treatment may be a surrogate for long-term survival patients with advanced melanoma.

**Clinical trial identification:**

Clinicaltrials.gov: NCT03605771

**Supplementary Information:**

The online version contains supplementary material available at 10.1007/s12094-025-04098-3.

## Introduction

Current options for the treatment of advanced melanoma include anti-PD-1-based immunotherapy and, in the case of BRAF-mutant disease, also targeted therapy [1]. These drugs produce objective responses in a significant proportion of patients and prolong survival. Median overall survival currently exceeds 2 years, but with wide margins: some patients still die within a few months after diagnosis, whereas others become long-term survivors [2].

Accurate prediction of benefit from standard therapy would allow proper selection of drugs and, in the case of resistant tumours, could lead to recommend the participation in clinical trials. For this reason, much effort has been devoted to find out predictors of response and long-term benefit in melanoma. Tumour burden, the pattern of metastatic spread, mutational burden, or gene signatures, among others, have been investigated [3, 4]. However, none of them are reliable enough to make decisions in clinical practice, so physicians usually consider clinical features to choose therapy and estimate prognosis. The matter remains an unmet need in the field of advanced melanoma.

GEM-1801 is a multi-cohort prospective study from the Spanish Multidisciplinary Melanoma Group. The study gathers information about tumour characteristics, local and systemic therapies, and outcomes in patients with stage III or stage IV melanoma. Over 1,000 patients with stage IV disease have been included, so we decided to interrogate the database about the clinical characteristics of patients who had become long-term survivors.

## Material and methods

### Study design

GEM-1801 (NCT03605771) is a multi-cohort prospective and retrospective study from the Spanish Multidisciplinary Melanoma Group that includes patients ≥18 years with resectable stage III melanoma or with unresectable stage III or stage IV melanoma. All patients provided a signed informed consent prior to inclusion. The study was carried out in 36 hospitals in Spain with a broad territory coverage.

The GEM-1801 study is designed in accordance with the International Ethical Guidelines for Biomedical Research Involving Human Subjects, Good Clinical Practice guidelines, the Declaration of Helsinki, 1964; local laws; SAS order 3470/2009; and Organic Law 3/2018, of December 5, on the Protection of Personal Data and guarantee of digital rights. The study was initially approved by the ethical committee from the Hospital Gregorio Marañón de Madrid (ref: 06/2018).

### Patient population

**Fig. 1:**
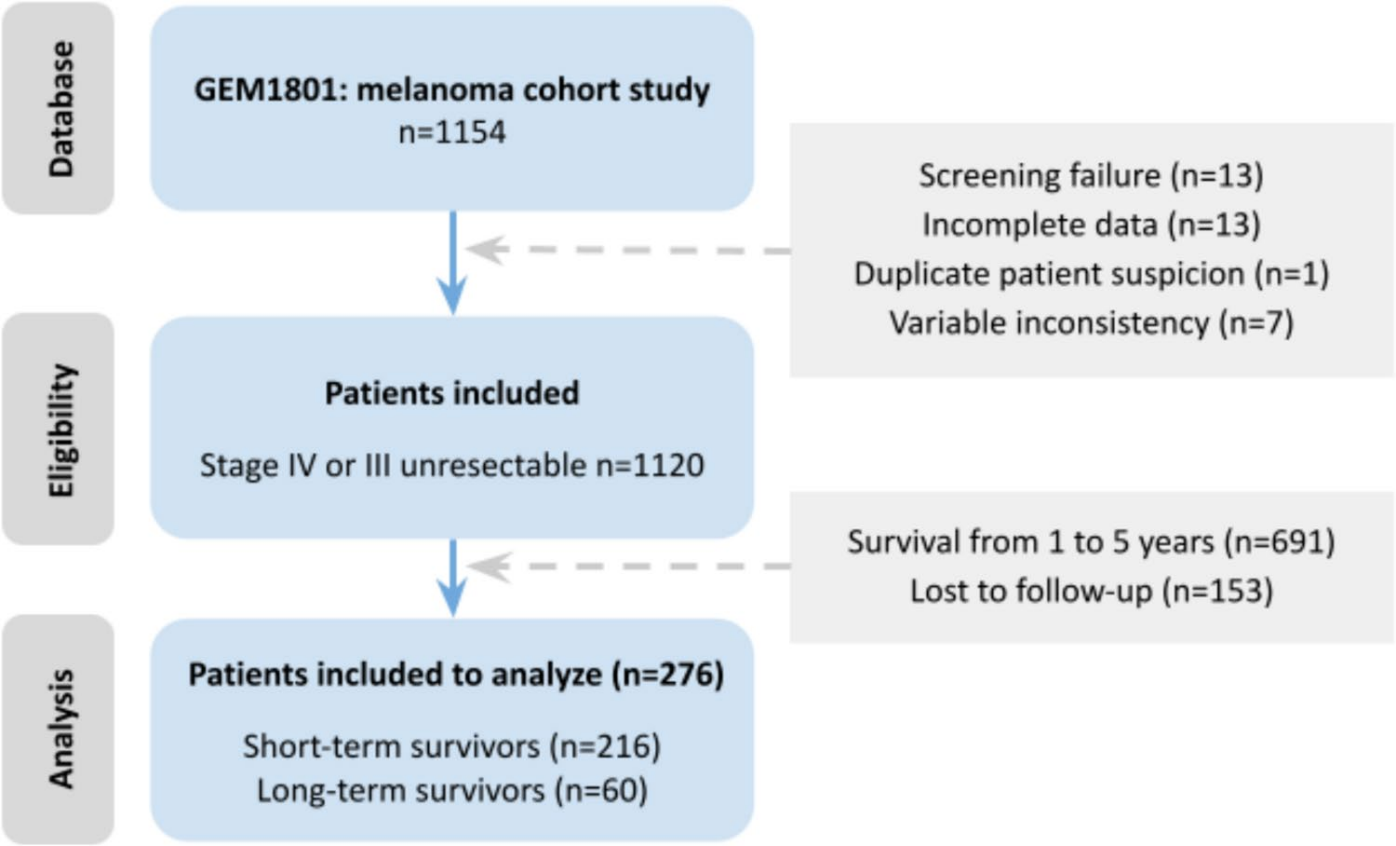
ESMO-GROW flow chart for the patient selection for analysis

Patients included in the GEM registry, ≥18 years old, both male and female, and with a diagnosis of melanoma after 2018. The database of GEM-1801 was searched to identify patients with unresectable stage III or stage IV melanoma who have survived at least 5 years since the diagnosis of this condition (Figure 1). Data from patients with the same diagnosis and surviving less than 1 year since diagnosis were also retrieved for comparison.

### Objectives and outcomes

The primary objective of this study is to describe the clinical features, therapies and outcomes of patients with advanced melanoma and long-term survival in the real-world in Spain. The exploratory objective was to evaluate the potential clinical prognostic factors that might help to identify long-term survivors in the real-world. The following items were retrieved: age, location of primary tumour, BRAF-mutation status, number of metastatic sites, location of metastases, level of lactate dehydrogenase (LDH), neutrophil to lymphocyte ratio (NLR), systemic therapy administered in the first and the second line treatment in the advanced setting. The Objective Response Rate (ORR) was defined as the percentage of patients achieving a complete response (CR) or partial response (PR) as best overall response; the progression-free survival (PFS) was defined as the time from the first-line treatment initiation to disease progression, death or loss to follow-up; and the OS was defined as the time from advanced disease diagnosis to death or loss to follow-up.

### Statistical analysis

The statistical analysis performed included descriptive statistics for quantitative variables and frequencies, percentages and confidence intervals for categorical variables, and the use of Kaplan-Meier method for time-to-event endpoints and survival analysis. The statistical tests performed were two-tailored and results with p<0.05 were marked as statistically significant. Statistical analyses were performed using R (version 3.6.3 [2020-02-29] “Holding the Windsock” R Foundation for Statistical Computing, Vienna, Austria).

## Results

### Patient selection

From August 2018 to December 2023, 1120 patients were included in the registry with a diagnosis of locally advanced unresectable or metastatic melanoma diagnosed between January 2018 and December 2023. Sixty of these patients (5%) remained alive after 5 years: they constitute the group of long-term survivors. The median overall survival was not reached at data cut-off (Supplementary Fig. 1a).

The comparison group was selected from the whole database. A total of 216 patients had a survival of less than 1 year (19%): they constitute the group of short-term survivors. The median overall survival was 7 months (Supplementary Fig. 1a). Figure 1 shows the flow chart for patient selection and Table 1 shows the clinical characteristics of both groups.

**Table 1:** Clinical characteristics of both groups

Characteristics		Long-term survivors n = 60	Short-term survivors n = 216	p value
Median age; years (range)		65 (31–90)	72 (22–95)	0.064^a^
Sex, n (%)	Female	34 (56.7)	78 (36.1)	0.005^b^
	Male	26 (43.3)	138 (63.9)	
Origin; n (%)	Cutaneous	45 (75.0)	134 (62.0)	0.401^d^
	Unknown	8 (13.3)	42 (19.4)	
	Acral	3 (5.0)	16 (7.4)	
	Mucosal	1 (1.7)	10 (4.6)	
	Uveal	1 (1.7)	10 (4.6)	
	Other	2 (3.3)	4 (1.9)	
Organs affected; n (%)	0–1	31 (51.7)	60 (27.8)	<0.001^c^
	≥2	29 (48.4)	156 (72.2)	
Metastasis location; n (%)	Brain	5 (8.3)	64 (29.6)	<0.001^b^
	Liver	11 (18.3)	71 (32.9)	0.0372^b^
	Bone	3 (5.0)	64 (29.6)	<0.001^b^
Number of metastasis locations; n (%)	0	1 (1.7)	1 (0.5)	<0.001^c^
	1	30 (50.0)	59 (27.3)	
	2	16 (26.7)	54 (26.4)	
	≥3	13 (21.7)	99 (45.8)	
NLR; n (%)	Normal	49 (81.7)	127 (58.8)	<0.001^b^
	Non normal	7 (11.7)	76 (35.2)	
LDH levels; n (%)	Normal	38 (63.3)	77 (35.6)	<0.001^b^
	Elevated (>1 ULN)	11(18.3)	98 (45.4)	
	NA	11 (18.3)	41 (19.0)	
BRAF status; n (%)	BRAF+	33 (55.0)	104 (48.1)	0.555^b^
	BRAF-	26 (43.3)	100 (46.3)	
	NA	1	12	
Comorbidities; n (%)	Yes	28 (46.7)	134 (62.0)	0.0382^b^
	No	32 (53.3)	82 (38.0)	
	Hypertension	18 (64.3)	100 (74.6)	0.3495^b^
	Dyslipemia	12 (42.9)	69 (51.5)	0.5336^b^

*NA* not available, *NLR* Neutrophil/Lymphocyte Ratio, *ULN* upper limit of normal

^a^Mann–Whitney test

^b^Fisher exact test

^c^Linear association test

^d^Pearson chi-squared test

### Clinical characteristics of long- and short-term survivors

Regarding the clinical characteristics of both groups, mucosal and uveal primaries were slightly more common in the group with short-term survival (4.6 vs 1.7% and 4.6% vs 1.7%, for mucosal and uveal, respectively). Women accounted for 57% of long-term survivors and 36% of short-term survivors (p = 0.0048, Fisher’s exact test). Short-term survivors had significantly more organs affected (45.8% with ≥3 organs affected vs 21.7%, p = 0.0001), stage M1C or M1D (63.4 vs 31.7%, p < 0.0001), abnormal neutrophil/lymphocyte ratio (37.5 vs 11.7%, p = 0.0001) and high-LDH levels (45.4 vs 18.3%, p < 0.0001) as compared with the long-term survival group (Table 1). The presence of brain (29.6 vs 8.3%), liver (32.9 vs 18.3%) and bone (29.6 vs 5.0%) lesions pertained a poor prognosis. The rate of comorbidities was also higher among patients with short survival (62.0 vs 46.7%, p = 0.0382), particularly hypertension (74.6 vs 64.3%) and dyslipidemia (51.5 vs 42.9%), but statistical significance was not reached for any particular condition.

Other factors did not significantly differ between short-term and long-term survivors: median age (71.6 vs 64.7 years, p = 0.0643), BRAF status (48.1 vs 55.0% with BRAF+, p = 0.5552), history of previous malignancy, cutaneous melanoma subtype (49.3 vs 66.7% superficial spread, 39.6 vs 31.1% nodular, and 6.7 vs 2.2% lentigo maligno, p = 0.2163), location (p = 0.5656) or T stage of primary melanoma (p = 0.8207) (Supplementary Table 1).

### Treatments

**Fig. 2:**
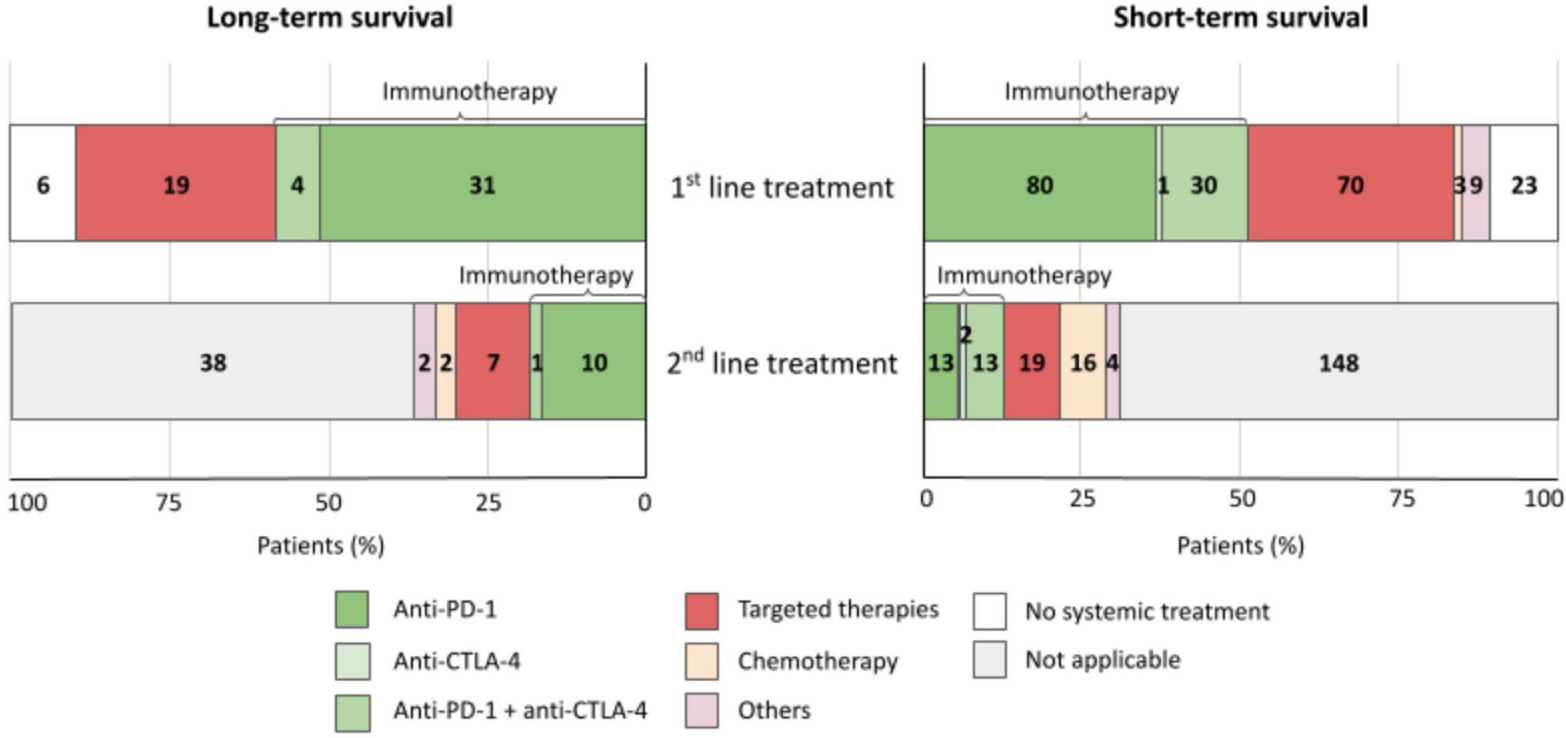
Stacked bar chart Treatments received by patients with long-term survival (left) and short-term survival (right) in the first-line and the second-line of systemic treatment. The numbers in the bars represent the number of patients receiving each treatment

**Fig. 3:**
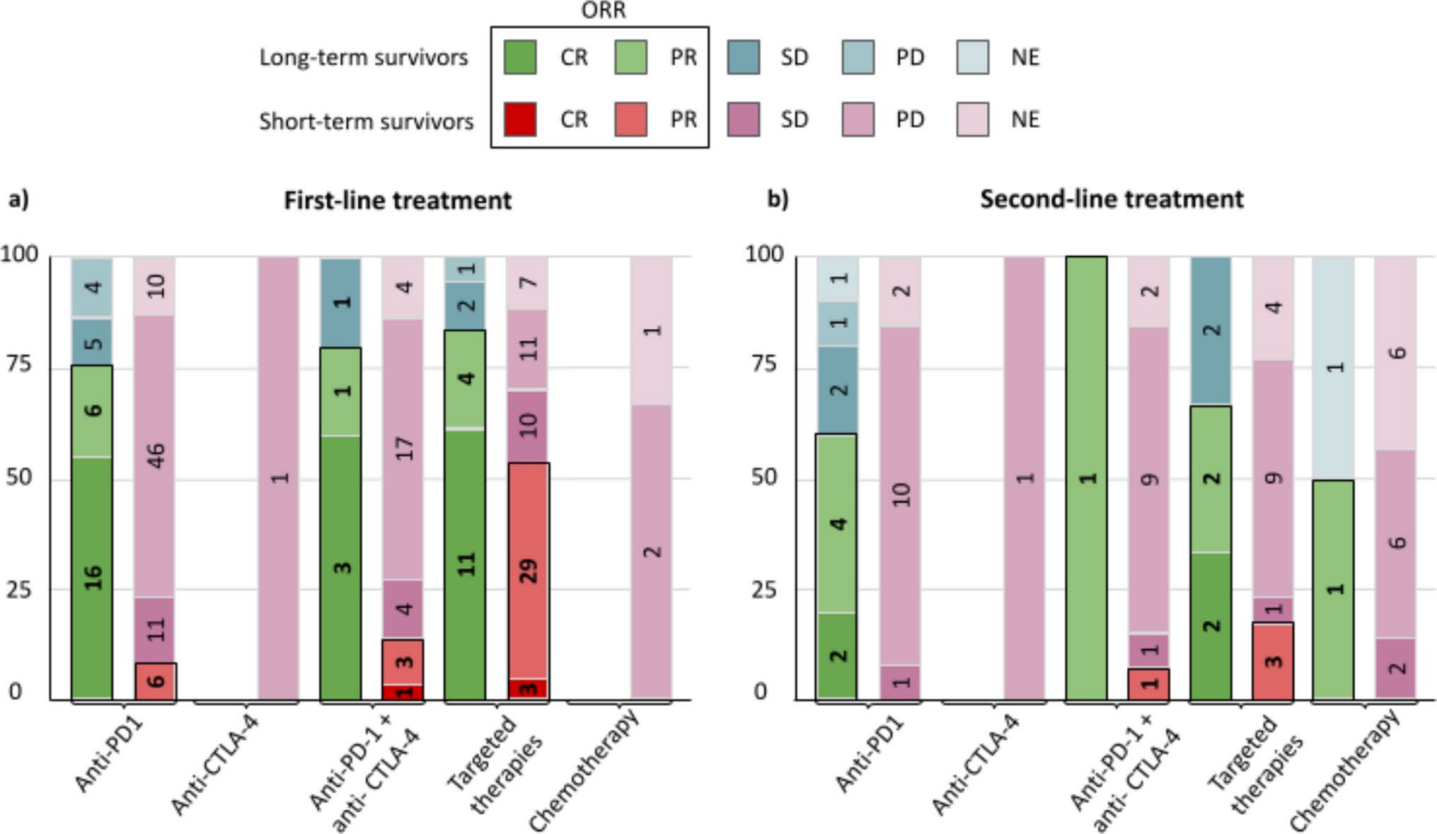
Stacked column chart for the Best Overall Response (BOR) and Objective Response Rate (highlighted in black) of long-term (green colors scale) and short-term (red color scale) patients receiving different types of systemic treatments in a) first-line treatment and b) second-line treatment. The BOR is expressed in terms of complete response (CR), partial response (PR), stable disease (SD), progression disease (PD) and not evaluable (NE). The numbers in each bar represent the number of patients achieving each response as BOR

Systemic therapy was administered in 54 patients with long-term survival (90%). The remaining 6 patients received local metastasis-directed therapies. The first line systemic therapy consisted of immunotherapy in 65% of patients and targeted therapy in 35% (Figure 2). Immunotherapy comprised single-agent anti-PD-1 in 86% of the cases and a combination of an anti-PD-1 and an anti-CTLA-4 in 14%. Targeted therapy was a combination of a BRAF inhibitor and a MEK inhibitor in all cases. The objective response rate was 76% for single-agent anti-PD-1, 80% for combination immunotherapy and 83% for targeted therapy (Figure 3a). Overall, 56% of patients in this group obtained a complete response and 20% a partial response with the systemic first line of treatment.

The PFS to first line therapy was not reached in the group of long-term survivors, with 77% of them being free of progression at 24 months (Supplementary Fig. 1b).

The second line treatment was administered to 22 long-term survivors (36%). It consisted of immunotherapy in 50% of these patients, targeted therapy in 32%, and chemotherapy in 9% (Figure 2). Immunotherapy comprised single-agent anti-PD-1 in 91% of the cases and a combination of an anti-PD-1 and an anti-CTLA-4 in 9%. The objective response rate was 60% for single-agent anti-PD-1, 100% for combination immunotherapy, 67% for targeted therapy, and 50% for chemotherapy (Figure 3b). Overall, 18% of patients in this group obtained a complete response and 36% a partial response with the second line of treatment.

Systemic therapy was administered in 193 patients with short-term survival (89%). Eleven patients received local metastasis-directed therapy and another 11 did not receive any treatment. First line systemic therapy among short-term survivors consisted of immunotherapy in 58% of patients, targeted therapy in 36%, and chemotherapy in 2%. Immunotherapy comprised single-agent anti-PD-1 in 70% of the cases, single-agent anti-CTLA-4 in 1%, and a combination of an anti-PD-1, and an anti-CTLA-4 in 29%. The objective response rate was 7% for single-agent anti-PD-1, 0 for anti-CTLA-4, 14% for combination immunotherapy, 53% for targeted therapy and 0 for chemotherapy (Figure 3a). Overall, 2% of patients in this group obtained a complete response and 19% a partial response with the first line of treatment.

The PFS to first line therapy was 3.4 months in the group with short-term survival (Supplementary Fig. 1b).

The second line treatment was administered to 67 short-term survivors (31%). It consisted of immunotherapy in 42% of these patients, targeted therapy in 28%, and chemotherapy in 24%. Immunotherapy comprised single-agent anti-PD-1 in 46% of the cases, single-agent anti-CTLA-4 in 7%, and a combination of an anti-PD-1 and an anti-CTLA-4 in 46%. The objective response rate was 0 for single-agent anti-PD-1, single-agent anti-CTLA-4, and chemotherapy, 8% for combination immunotherapy, and 18% for targeted therapy (figure 3b). Overall, 6% of patients in this group obtained a partial response with the second line of treatment.

## Discussion

This study retrieved clinical information on patients with advanced melanoma who survived longer than 5 years. Patients participated in GEM-1801, a multi-cohort prospective study by the Spanish Multidisciplinary Melanoma Group (GEM). The results show that response to systemic therapy strongly correlates with long-term survival. Other known prognostic factors such as female gender, a limited number of metastatic locations and normal LDH level were also more common among survivors. On the contrary, age, BRAF mutational status, tumour subtype and anatomic location of the primary tumour did not correlate with outcome.

The relation between the obtention of a response and survival is well documented with anti-PD-1-based therapy. In Checkmate 067, which compared nivolumab or nivolumab plus ipilimumab versus ipilimumab, long-term melanoma-specific survival exceeded 70% in patients who received an anti-PD-1 antibody and achieved an objective response [5]. Similar results were reported in Keynote 006, comparing pembrolizumab versus ipilimumab [6]. The Dutch registry, however, shows that the outcome was less favourable in the case of a partial response [7]. There is also some information about targeted therapy in this regard. A pooled analysis of trials with dabrafenib plus trametinib showed that the 5-year survival rate was 71% in patients who attained a complete response but dropped to 32% following a partial response [8]. In our series, most patients surviving 5 years had a complete response.

It is important to note that approximately one third of these patients required a second line of therapy. In most cases, they had BRAF-mutant disease where immunotherapy was substituted by targeted therapy or vice versa. Availability of an effective second line may explain why outcome in BRAF-mutant disease is slightly superior to BRAF-wild type disease in some recent studies [5]. On the contrary, in our group with short survival there was a predominance of BRAF-wild type tumours, and chemotherapy was the preferred second line following immunotherapy.

The 5-year survival rate in GEM-1801 was 26%, which is inferior to that reported in pivotal trials of immunotherapy [5, 6] and similar to that of dabrafenib and trametinib [8]. Our patients started therapy before October 2023, when the long-term superiority of combination immunotherapy over targeted therapy had not yet been demonstrated [9]. Targeted therapy was favoured at that time in our country, therefore most patients with BRAF-mutant disease received targeted therapy. Response rate in patients with long-term survival was similar for single-agent anti-PD1 and anti-PD1 plus anti-CTLA4. This suggests that patients with favourable prognostic factors may not need combination immunotherapy. However, this conclusion should be considered cautiously, as our series is relatively small, and information was not available for some prognostic factors such as PD-L1 expression or the appearance of immune-mediated side effects.

The correlation between response and long-term survival may influence the general strategy of treatment for advanced melanoma. For instance, patients obtaining only a partial response or stable disease could undergo frequent follow-up with either imaging techniques or liquid biopsy to detect early progression and initiate salvage therapy as soon as possible. Participation in clinical trials should be encouraged in this context, considering the low response rate that we observed with second line therapy.

The correlation of tumour burden and LDH levels with outcome has been previously described with current therapeutic options. In a pooled-analysis of dabrafenib and trametinib, a regression-tree analysis included these two factors to predict response, progression-free survival and overall survival [10]. In the pivotal trial of pembrolizumab, patients with elevated LDH had a reduced 10-year survival rate [11].

Our study has some limitations. First, as a cohort study (and not a formal registry) it represents a fraction of all patients diagnosed with advanced melanoma in that period. This could have led to a selection bias of patients with poor prognosis treated in large centres. Second, the population was small to draw conclusions in subgroup analysis. Finally, response was estimated by local physicians, and there was not a central radiological review.

As a conclusion, response to either first or second line of therapy may be a surrogate marker for long-term survival in patients with advanced melanoma. On the contrary, lack of response to first line, particularly In patients with BRAF wild-type disease remains an unmet need in the field.

## Supplementary Information

Below is the link to the electronic supplementary material.

Supplementary file1 (DOCX 112 KB)

## Data Availability

Data are recorded as part of GEM 1801, a multicohort prospective study by the Spanish Melanoma Group (GEM)
