# Supplementary material for: Response rate and long-term survival in patients with advanced melanoma: data from the prospective cohort study gem-1801
Source: Clin Transl Oncol. 2025 Nov 28;28(5):1807–14. doi: 10.1007/s12094-025-04098-3 (PMC13099804; doi:10.1007/s12094-025-04098-3)

**SUPPLEMENTARY MATERIAL**

**Supplementary table 1.** Additional patients’ baseline characteristics.

| **Characteristics** | | **Long-term survivors**  **n=60** | **Short-term survivors**  **n = 216** | **p-value** |
| --- | --- | --- | --- | --- |
| **Cutaneous histological subtype; n (%)** | Superficial spread | 30 (66.7) | 66 (49.3) | 0.216^a^ |
|  | Nodular | 14 (31.1) | 53 (39.6) |  |
|  | Lentigo maligna | 1 (2.2) | 9 (6.7) |  |
|  | Not done | 0 (0.0) | 3 (2.2) |  |
|  | Other | 0 (0.0) | 3 (2.2) |  |
| **Location; n (%)** | Trunk | 17 (33.3) | 61 (37.2) | 0.566^a^ |
|  | Extremities | 17 (33.3) | 32 (19.5) |  |
|  | Head | 9 (17.6) | 28 (17.1) |  |
|  | Feet | 3 (5.9) | 14 (8.5) |  |
|  | Digestive | 1 (2.0) | 3 (1.8) |  |
|  | Hands | 0 (0.0) | 2 (1.2) |  |
|  | Genitourinary | 0 (0.0) | 1 (0.6) |  |
|  | Oral | 0 (0.0) | 1 (0.6) |  |
|  | Others | 3 (5.9) | 21 (12.8) |  |
|  | NA | 9 | 52 |  |
|  | Missing | 1 | 1 |  |
| **Primary T stage; n (%)** | Tx | 5 (8.3) | 23 (10.6) | 0.8207^b^ |
|  | T0 | 9 (15.0) | 41 (19.0) |  |
|  | T1 | 0 (0.0) | 1 (0.5) |  |
|  | T1a | 3 (5.0) | 7 (3.2) |  |
|  | T1b | 3 (5.0) | 7 (3.2) |  |
|  | T2 | 0 (0.0) | 6 (2.8) |  |
|  | T2a | 6 (10.0) | 21 (9.7) |  |
|  | T2b | 3 (5.0) | 8 (3.7) |  |
|  | T3 | 0 (0.0) | 1 (0.5) |  |
|  | T3a | 8 (13.3) | 7 (3.2) |  |
|  | T3b | 6 (10.0) | 23 (10.6) |  |
|  | T4 | 2 (3.3) | 0 (0.0) |  |
|  | T4a | 7 (11.7) | 20 (9.3) |  |
|  | T4b | 8 (13.3) | 51 (23.6) |  |
| **History of previous malignancy; n (%)** | No | 44 (73.3) | 174 (80.6) | 0.4151^a^ |
|  | Cutaneous non melanoma | 1 (1.7) | 5 (2.3) |  |
|  | Others | 13 (21.7) | 32 (14.8) |  |
|  | Missing | 2 | 5 |  |

^a^Pearson chi-squared test, ^b^linear-by-linear association test

**SUPPLEMENTARY MATERIAL.**

**Supplementary figure 1.** Kaplan-Meier curves for patients with short-term and long-term survival for the **a)** overall survival and **b)** progression-free survival.


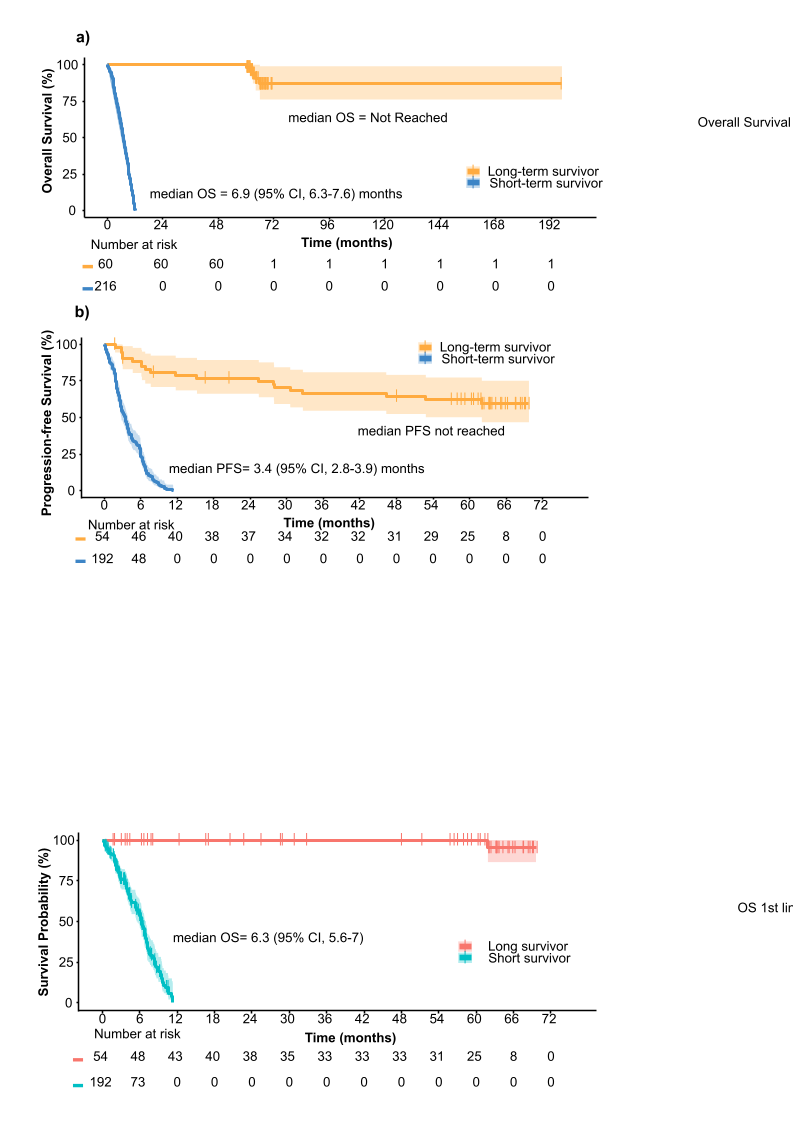

Supplement: Supplementary file 1 — Supplementary file1 (DOCX 112 KB) [file 12094_2025_4098_MOESM1_ESM.docx]
